# Exploring Implicit Emotional Associations With Death in Patients With Current Suicidal Ideation: Results From Novel Attitude Implicit Association Tests for Suicide

**DOI:** 10.1111/sltb.70047

**Published:** 2025-09-07

**Authors:** L. M. Aschenbrenner, A. Frei, D. Knapp, T. Forkmann, D. Schreiber, H. Glaesmer, J. Brüdern, M. Stein, S. Walther, A. Gysin‐Maillart

**Affiliations:** ^1^ Translational Research Centre University Hospital of Psychiatry and Psychotherapy, University of Bern Bern Switzerland; ^2^ Graduate School for Health Sciences, Faculty of Medicine University of Bern Bern Switzerland; ^3^ Department of Clinical Psychology University of Duisburg‐Essen Essen Germany; ^4^ Department of Medical Psychology and Medical Sociology University of Leipzig Leipzig Germany; ^5^ Department of Clinical Psychology and Psychotherapy University of Bern Bern Switzerland; ^6^ Department of Psychiatry, Psychosomatics and Psychotherapy, Center of Mental Health University Hospital of Würzburg Würzburg Germany; ^7^ Department of Clinical Sciences Lund University Lund Sweden

**Keywords:** implicit cognitive processes, suicidal behavior, suicidal ideation, suicidal mode, suicide risk assessment

## Abstract

**Introduction:**

Assessing suicide risk in clinical settings is challenging, as conventional self‐report scales have limited predictive validity. The Death Implicit Association Test (D‐IAT) was developed to explore implicit associations related to death and the self‐concept. However, it omits the emotional association with death that may be crucial in suicide risk assessment. This cross‐sectional study on individuals with current suicidal ideation integrates the implicit emotional association with death into the conventional D‐IAT.

**Methods:**

We aimed to explore whether patients with current suicidal ideation exhibit more positive implicit emotional associations with death compared to a clinical control group without current suicidal ideation and lifetime suicidal behavior (total *N* = 182). We employed the standard identity D‐IAT (D‐IAT_me/not me_) and two novel attitude D‐IAT versions (D‐IAT_I like/I don’t like_, D‐IAT_pleasant/unpleasant_). Furthermore, we compared all versions regarding their predictive and discriminative validity and analyzed correlations between implicit associations and self‐reported suicidal ideation.

**Results:**

In the D‐IAT_me/not me_ and D‐IAT_I like/I don’t like_ version, patients with current suicidal ideation exhibited weaker negative associations with death compared to clinical controls. The D‐IAT_pleasant/unpleasant_ did not yield a group difference. The D‐IAT_me/not me_ showed superior performance in predictive validity and a similar performance in discriminative validity as the D‐IAT_I like/I don’t like_. These two versions correlated positively with self‐reported current suicidal ideation. In the D‐IAT_pleasant/unpleasant_, no such correlation was found.

**Discussion:**

Our findings substantiate the validity and reliability of the identity D‐IAT and suggest the D‐IAT_I like/I don’t like_ as a potential complementary attitude variant with personalized categories. Incorporating implicit emotional associations when working with suicidal patients could enhance the evaluation and treatment of individuals at risk of suicide. Further investigation is warranted to gain a more comprehensive understanding of these relationships.

## 1 Introduction

Each year, more than 700,000 people die by suicide worldwide (World Health Organization 2021). Assessing suicide risk, detecting, predicting, and preventing suicidal thoughts and behavior (STBs) pose significant clinical challenges. One reason may be the high fluctuation (Hallensleben, 2018) and varying intensity (Kleiman et al. 2017; Nock, Hwang, et al. 2010, Nock, Park, et al. 2010) of suicidal ideation between individuals. Patients experience suicidal ideation with variable frequency and intensity. Furthermore, patients may intentionally conceal suicidal thoughts (Carter et al. 2017) or may not have sufficient awareness to access their suicidal thoughts introspectively (Wilson et al. 2000). Therapeutic communication tailored to the individual person is therefore important for assessing the risk of suicidal behavior (King et al. 1997). However, communication between the therapist and patient often is problematic. Suicidal patients require special communication, which is based on secure relationships in which patients can share their feelings of pain and shame (Gysin‐Maillart et al. 2016). Often, these conditions are lacking, as many individuals who attempted suicide had contact with medical professionals shortly before the attempt but were not able to communicate their suicidal thoughts (Nock and Banaji 2007). Therefore, a complementary approach to understanding and assessing suicide risk is needed.

It may be possible to supplement *explicit* verbal self‐reporting (e.g., “I want to die”) by applying *implicit* assessment tools, such as the *Implicit Association Test* (IAT). The IAT aims to capture implicit associations or attitudes through a computer‐based task in which words must be assigned to contrasting constructs. Based on reaction times (RTs), this setup allows the calculation of implicit association strength between the constructs, assuming that responses to stimuli of the IAT are largely automatic and receive little conscious awareness (Greenwald and Banaji 1995). Several studies have investigated whether suicide risk can be assessed by using the *Death IAT* (D‐IAT; Nock, Park, et al. 2010; Rath et al. 2018). The D‐IAT contains two bipolar target categories, “death” and “life”. It measures the differential strength of implicit associations between “death” and “me” and between “life” and “me”, that is, implicit self‐death associations (“death‐identity bias”; Hussey et al. 2016), driven by the hypothesis that patients with STBs associate themselves more strongly with death than those without STBs.

In the context of the *Ideation‐to‐Action Framework* proposed by Klonsky and May (2014), understanding implicit associations with death may be critical to unraveling the unconscious processes underlying the transition from suicidal ideation to behavior. While implicit associations are not explicitly addressed in the traditional framework, they can be conceptually integrated. In the transition from thinking about suicide to actively planning and preparing for suicidal behaviors, underlying implicit processes may influence the transition from suicidal ideation to suicidal behavior (Brüdern et al. 2022).

Studies using the standard D‐IAT, that is, the identity version using “me” and “not me”, have yielded inconsistent results. Harrison et al. (2020) found that most individuals exhibited more robust implicit associations with life. Several studies have indicated significant differences in the association strengths between attempters and non‐attempters. Harrison et al. (2014) found that the D‐IAT predicted five out of six suicide risk indicators mediated by survival and coping beliefs. Millner et al. (2018) reported that the Brief D‐IAT effectively distinguished past‐year and lifetime attempters. Podlogar et al. (2020) found that D‐IAT scores were related to the severity of past suicidal behavior, moderated by attempt history and wish to live. Wang et al. (2022) demonstrated that the D‐IAT differentiated patients with and without suicide attempts, with stronger correlations in female patients. Conversely, other studies have not found distinctions in D‐IAT scores between attempters and non‐attempters overall (Barnes et al. 2017; Dickstein et al. 2015; Millner et al. 2019; Rath et al. 2021; Tello et al. 2020). Longitudinally, Scheunemann et al. (2021) found that within an 18‐month follow‐up, implicit measures, including the D‐IAT, had an additional predictive value for suicidal ideation beyond self‐report measures at baseline. However, the implicit measures did not demonstrate predictive validity for suicide plans or attempts. Despite these mixed results, meta‐analytic evidence demonstrated that the D‐IAT predicts past and future suicidal behavior, albeit with a weak effect size (Sohn et al. 2021). The observed effect heterogeneity can be explained by differences in study settings, such as acute care versus community settings.

Several studies from social psychology and addiction research have used an attitude version of the IAT to investigate implicit emotional associations. Greenwald et al. (2009) showed that the validity of the attitude version IAT measuring concept‐valence associations in predicting behavioral, judgemental, and physiological measures significantly surpassed that of self‐report measures. For example, individuals with self‐reported high alcohol consumption showed stronger associations between alcoholic drinks and implicit positive affective categories compared to non‐alcoholic drinks (Houben et al. 2010; Houben and Wiers 2007; Olson and Fazio 2004). In particular, the use of personalized affective categories (“I like” and “I don't like”) has been shown to reduce the influence of extrapersonal associations, that is, socially desirable responding, on the IAT and has increased the validity of the test (Houben and Wiers 2007). Olson and Fazio (2004) highlighted that the traditional IAT labels, such as “good” and “bad”, can encourage participants to respond based on societal normative implications rather than personal attitudes and preferences. Nock and Banaji (2007) have examined the implicit emotional assessment in the context of self‐injurious thoughts using the *Self‐Injury IAT* (SI‐IAT). Their study showed stronger discriminant and predictive effect sizes for the identity version compared to the attitude version. However, to the best of our knowledge, there exist no further death‐ or suicide‐related IAT studies using attitude stimuli. While the standard identity D‐IAT procedure has demonstrated validity, albeit with mixed findings and varying predictive value across studies, exploring implicit emotional associations with death, that is, “death‐evaluation biases” (Hussey et al. 2016), which refer to cognitive and affective biased evaluations of death, could present a promising direction to systematically explore a more comprehensive picture of implicit dimensions in suicidality.

In the current study, our aim is to investigate the implicit attitude assessments in suicidality. We are particularly interested in exploring the implicit emotional associations with death among individuals experiencing current suicidal ideation. Based on the concept of the “death‐evaluation bias”, we have developed two novel versions of the D‐IAT, incorporating affective categories designed to measure implicit emotional responses in relation to the traditional bipolar categories of “death” and “life”.

Our primary aim is to examine whether patients with current suicidal ideation show different implicit emotional associations with death than a clinical control group without suicidal ideation or lifetime suicidal behavior. (1) We hypothesize that patients with current suicidal ideation will show a stronger association between death and themselves, as measured in the standard D‐IAT. (2) Additionally, we hypothesize that patients with suicidal ideation will show stronger associations between death and positive affective categories on the novel attitude‐based D‐IAT versions compared to the clinical controls. (3) We aim to compare the novel attitude‐based versions of the D‐IAT with the standard identity‐based version proposed by Nock, Park, et al. (2010). Specifically, we will explore whether there are differences in the predictive, discriminant, and convergent validity of these versions. (4) Finally, we will explore correlations between implicit associations with death, as assessed by all three D‐IAT versions, and explicit suicidal ideation across the two subgroups. We hypothesize that stronger implicit associations with death will be linked to higher levels of explicit suicidal ideation.

## 2 Methods

### 2.1 Sample

We conducted a cross‐sectional analysis of *N* = 182 participants, aged 18 to 64 years (*M* = 34.82, SD = 13.28), of which 56% were female (*n* = 101). The sample consisted of two groups: psychiatric in‐patients currently experiencing suicidal ideation (suicide ideators; *n* = 91) and clinical controls, who were in‐patients (*n* = 91) neither reporting current suicidal ideation nor lifetime suicidal behavior. The suicide ideators group consisted of patients who reported suicidal ideation within the prior 7 days, including the test day, based on their responses to items 4 and 5 of the German version of the *Beck Scale for Suicide Ideation* (BSSI; Beck and Steer 1993; Kliem and Brähler 2015). Of these patients, *n* = 39 (43%) reported having made a suicide attempt in the past. The clinical control group included patients who were receiving treatment for any psychiatric disorder within an in‐patient clinical setting. The distribution of key demographic characteristics and clinical variables of the two groups is shown in Table 1. In the total sample, the most common disorders were mood (*n* = 138; 76%) and anxiety disorders (*n* = 104; 57%). Among the cohort, 60% (*n* = 110) qualified for multiple diagnoses. Baseline differences in diagnostic variables were found in mood disorders and the obsessive‐compulsive spectrum.

**TABLE 1:** Baseline characteristics of participants.

	Suicide ideators (*n* = 91)	Clinical controls (*n* = 91)	Test statistic	*p* value
Gender, female/male and others (*n*; %)	55 (60)/36 (40)	46 (51)/45 (49)	2.32^a^	0.314
Age, years (*M*, SD)	31.3 (11.7)	38.4 (13.9)	2854.00^b^	< 0.001
Diagnosis (DSM‐IV) (*n*)				
Mood disorders	85	53	30.69^a^	< 0.001
Anxiety disorders	57	47	2.24^a^	0.134
Obsessive‐compulsive spectrum	44	27	6.67^a^	0.010
Trauma‐related disorders	17	15	0.15^a^	0.697
BSSI Suicidal Ideation Mean (SD)	17.1 (6.2)	0.4 (0.8)	0.00^b^	< 0.001
BSSI Suicide Attempts (*n*, %)			50.71^a^	< 0.001
No previous attempt	52 (57)	91 (100)		
One previous attempt	16 (18)	0 (0)		
> Two previous attempts	23 (25)	0 (0)		

*Note:* M = mean; SD = standard deviation; diagnoses recorded with the M.I.N.I. (Ackenheil et al. 1999; Sheehan et al. 1998); explicit variables assessed with the BSSI = Beck Scale for Suicide Ideation (Beck and Steer 1993; Kliem and Brähler 2015).

^a^ Chi‐square test.

^b^ Mann–Whitney *U* test.

**FIGURE 1:**
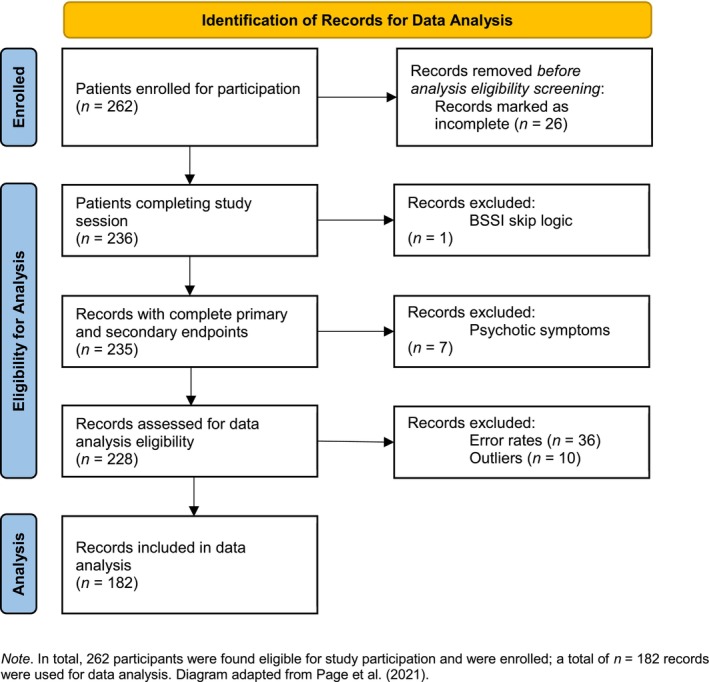
Flow diagram of record identification for data analysis. In total, 262 participants were found eligible for study participation and were enrolled; a total of *n* = 182 records were used for data analysis. Diagram adapted from Page et al. (2021).

Exclusion criteria for both groups were current psychotic symptoms, age below 18 and above 65 years, inability to speak or write German fluently, and cognitive impairments. Eighty data sets were excluded from the initially enrolled sample of *N* = 262 for analysis, see Figure 1: 36 cases (14%) with excessive error rates in the D‐IAT (see *Procedure* section), 26 participants (10%) who were unable to complete the study session due to problems with concentration, migraine, or poor eyesight, 7 patients (3%) who were excluded due to psychotic symptoms according to the *Mini International Neuropsychiatric Interview for Adults* (M.I.N.I., Ackenheil et al. 1999; Sheehan et al. 1998), and 1 instance (0.4%) with incorrect implementation of the skip logic in the BSSI. We further excluded 10 outlier datasets (4%) from our analysis (see *Statistical Procedures* section).

### 2.2 Procedure

Data was collected at the University Hospital for Psychiatry and Psychotherapy in Bern (UPD) from May 2020 to February 2024. The study protocol was approved by the Ethics Committee (KEK 2019‐01410; ClinicalTrials.gov ID NCT04585802) and was conducted in accordance with the Declaration of Helsinki (World Medical Association 2001). Participants provided written informed consent. Study participation took approximately 1.5 h and was structured into three phases: completion of the D‐IAT, completion of questionnaires, and the structured diagnostic interview M.I.N.I.

The study session started with the three D‐IAT versions on a computer with screen sizes ranging between 15.6 in. and 17.3 in. using *Presentation* software Version 20.3 from *Neurobehavioral Systems*. The order of the three D‐IAT versions was randomized to minimize potential sequence effects.

Following the completion of the D‐IATs, participants proceeded to fill out demographic and clinical self‐report questionnaires.

Finally, participants underwent a clinical assessment using the M.I.N.I. (Ackenheil et al. 1999; Sheehan et al. 1998). This structured diagnostic interview served the dual purpose of confirming eligibility for group assignment and assessing Axis I disorders according to DSM‐IV criteria.

Trained research team members administered all phases of the study. The participants received no monetary compensation for their study participation.

## 3 Measurements

The English version of the *D‐IAT* (Nock, Park, et al. 2010) was translated into German (Rath et al. 2018) according to the guidelines of the ISPOR Task Force for Translation and Cultural Adaptation (Wild et al. 2005). All categories and stimuli used in the three D‐IAT versions were presented in German. Each D‐IAT task consisted of seven blocks in which participants were presented with words in the centre of the screen. The stimuli had to be classified into two predefined categories displayed on the upper half of the screen by pressing the two response keys “E” or “I” on the laptop keyboard. Based on Nock, Park, et al. (2010) and Rath et al. (2018), all D‐IATs used contained two bipolar target categories, “Tod” (“death”) and “Leben” (“life”). “Tod” included the stimuli “Suizid”, “sterben”, “Beerdigung”, “leblos”, and “verstorben” (“suicide”, “die”, “funeral”, “lifeless”, and “deceased”); “Leben” included “lebendig”, “leben”, “gedeihen”, “überleben”, and “atmend” (“alive”, “living”, “thriving”, “surviving”, and “breathing”). The stimuli used in the identity D‐IAT_Ich/Nicht‐Ich_ (D‐IAT_me/not me_) contained those stimuli used by Nock, Park, et al. (2010) and Rath et al. (2018) (see Table 2). The affective categories in the two attitude D‐IATs replaced the attribute categories “Ich” (“me”) and “Nicht‐Ich” (“not me”) from the D‐IAT_Ich/Nicht‐Ich_ (D‐IAT_me/not me_). These were named “angenehm” (“pleasant”) and “unangenehm” (“unpleasant”) in one version and “Ich mag” (“I like”) and “Ich mag nicht” (“I don't like”) in the other version. The stimuli used in the affective categories included words intended to evoke positive and negative emotions (see Tables 3 and 4). The two attitude D‐IAT versions did not differ regarding the word stimuli used but in the labelling of the affective categories. RTs for correct categorisations were recorded. Based on differences in RTs, we computed D‐values using the original scoring algorithm of the English version (Nock, Park, et al. 2010) to assess the strength and direction of participants' implicit associations with death. The more positive the D‐values, the stronger the implicit association with death.

**TABLE 2:** Structure of the identity D‐IAT_Ich/Nicht‐Ich_ (D‐IAT_me/not me_).

Order	Block	1	2	3 & 4	5	6 & 7
		Practice		Experimental	Practice	Experimental
A	Left	Tod	Nicht‐Ich	Tod/Nicht‐Ich	Leben	Leben/Nicht‐Ich
	Right	Leben	Ich	Leben/Ich	Tod	Tod/Ich
B	Left	Leben	Nicht‐Ich	Leben/Nicht‐Ich	Tod	Tod/Nicht‐Ich
	Right	Tod	Ich	Tod/Ich	Leben	Leben/Ich

*Note:* The identity version contains the attribute categories “Ich” (“me”) and “Nicht‐Ich” (“not me”). “Ich” (“me”) contains “Ich selbst”, “mein”, “meins”, “mich”, and “selbst” (“myself”, “my”, “mine”, “I”, “self”); “Nicht‐Ich” (“not me”) contains “ihnen”, “sie”, “ihres”, “ihr”, and “andere” (“them”, “they”, “theirs”, “their”, “other”) (see Nock, Park, et al. 2010; Rath et al. 2018).

**TABLE 3:** Structure of the attitude D‐IAT_angenehm/unangenehm_ (D‐IAT_pleasant/unpleasant_).

Order	Block	1	2	3 & 4	5	6 & 7
		Practice		Experimental	Practice	Experimental
A	Left	Tod	Unangenehm	Tod/unangenehm	Leben	Leben/unangenehm
	Right	Leben	Angenehm	Leben/angenehm	Tod	Tod/angenehm
B	Left	Leben	Unangenehm	Leben/unangenehm	Tod	Tod/unangenehm
	Right	Tod	Angenehm	Tod/angenehm	Leben	Leben/angenehm

*Note:* The target categories “Tod” (“death”) and “Leben” (“life”) contain the same stimuli as the identity version (see Nock, Park, et al. 2010; Rath et al. 2018). The affective category “unangenehm” (“unpleasant”) contains the stimuli “Trauer”, “Krieg”, “Leid”, “Schmerz”, and “Krankheit” (“sadness”, “war”, “suffering”, “pain”, “illness”); the affective category “angenehm” (“pleasant”) includes the stimuli “Liebe”, “Freiheit”, “Wärme”, “Frieden”, and “Sicherheit” (“love”, “freedom”, “warmth”, “peace”, “security”).

**TABLE 4:** Structure of the attitude D‐IAT_Ich mag/Ich mag nicht_ (D‐IAT_I like/I don’t like_).

Order	Block	1	2	3 & 4	5	6 & 7
		Practice		Experimental	Practice	Experimental
A	Left	Tod	Ich mag nicht	Tod/Ich mag nicht	Leben	Leben/Ich mag nicht
	Right	Leben	Ich mag	Leben/Ich mag	Tod	Tod/Ich mag
B	Left	Leben	Ich mag nicht	Leben/Ich mag nicht	Tod	Tod/Ich mag nicht
	Right	Tod	Ich mag	Tod/Ich mag	Leben	Leben/Ich mag

*Note:* The target categories “Tod” (“death”) and “Leben” (“life”) contain the same stimuli as the identity version (see Nock, Park, et al. 2010; Rath et al. 2018). The affective category “Ich mag nicht” (“I don't like”) contains the stimuli “Trauer”, “Krieg”, “Leid”, “Schmerz”, and “Krankheit” (“sadness”, “war”, “suffering”, “pain”, “illness”); the affective category “Ich mag” (“I like”) includes the stimuli “Liebe”, “Freiheit”, “Wärme”, “Frieden”, and “Sicherheit” (“love”, “freedom”, “warmth”, “peace”, “security”).

For one‐half of the participants, the “Leben”/”Ich” (“life”/”me”), “Leben”/”angenehm” (“life”/”pleasant”), and “Leben”/”Ich mag” (“life”/”I like”) blocks were presented first, and for the other half, the “Tod”/”Ich” (“death”/”me”), “Tod”/”angenehm” (“death”/”pleasant”), and “Tod”/”Ich mag” (“death”/”I like”) blocks were presented first. This allocation was randomized. Following the original scoring method, participants with over 10% RTs below 300 ms were excluded. Likewise, participants with error rates above 30% in the four experimental blocks 3, 4, 6, and 7 (or 40% per block) were excluded. RTs above 10,000 ms were included in the calculation as missing values. RTs for error trials were considered longer RTs until the correct button was pressed to calculate the D‐value.

The *BSSI* (Beck and Steer 1993; German translation: Kliem and Brähler 2015) serves as a 21‐item self‐report tool designed to assess the current intensity of a patient's suicidal ideation. Initial screening for suicidal ideation involves the first five BSSI items. If the patient chooses the null statements on both items four and five, which explore a person's desire to die (item four) and to save one's life in a life‐threatening situation (item five), they can skip the subsequent 14 items, which explore specific details about the respondent's suicide plans and attitudes. Each item rating ranges from zero to two. The severity of suicidal ideation is quantified by summing the scores of the first 19 items, with a total score ranging from 0 to 38. Qualitative items 20 and 21, which refer to previous suicidal behavior, are excluded from the total score. For the group assignment, we used items four and five. Patients with a response higher than 0 on either item four or five were assigned to the suicide ideators group. The German version of the BSSI has demonstrated good reliability and validity (Kliem et al. 2017). In this study, Cronbach's alpha yielded a coefficient of *α* = 0.85, indicating high internal consistency among the items.

The *M.I.N.I*. (Ackenheil et al. 1999; Sheehan et al. 1998) is a structured diagnostic interview designed to screen participants for Axis I disorders according to DSM‐IV criteria and to assess suicide risk. The German version of the M.I.N.I. (Ackenheil et al. 1999) was used to cross‐validate group eligibility based on “Modul C. Suicidality”. A “yes” to the module's diagnostic field, which suggests a current suicide risk, led to the exclusion of control subjects from their group.

We collected sociodemographic data (DEMO; Gysin et al., 2016, revised 2019), including age, gender, marital status, recent self‐harming behaviors, and suicidal ideation/preparations/behaviors within the past 6 months.

## 4 Statistical Procedures

The collected data underwent an extensive screening process to ensure compliance with the assumptions required for statistical analysis, such as normality, homoscedasticity, identification of outliers, and data completeness. All statistical analyses were conducted using SPSS 29.0.

The a priori power analysis indicated a sample size of *N* = 179 to detect a medium effect size (*d* = 0.25) with a power of 0.8 at a significance level of 0.05 for the repeated measures analysis of variance (RM‐ANOVA) testing group differences. Thus, the study was sufficiently powered.

Prior to data analysis, we checked for outliers in our dataset following established procedures (Polit, 2010). Having a sample size of *n* ≥ 50, we performed a Kolmogorov–Smirnov test (Mishra et al. 2019) to determine the distribution of D‐values across all versions. The distribution of the D‐IAT_angenehm/unangenehm_ deviated significantly from normality (*W*(192) = 0.08, *p* = 0.008). Hence, we identified outliers based on the interquartile range (IQR) derived from the D‐values of each D‐IAT. Specifically, data points lying between 1.5 and 3 times beyond the length of the IQR were considered outliers, in line with standard practices (Field and Miles 2010). Given the conservative outlier management through deletion (Mowbray et al., 2018), outliers meeting these criteria were systematically removed from the dataset (see also Figure 1).

To test our hypothesis that suicide ideators and clinical controls would differ in the three D‐IAT versions, we conducted a RM‐ANOVA with “group” as a between‐subject factor (suicide ideators vs. clinical controls) and “iat_version” as a within‐subject factor (D‐IAT_Ich/Ich‐Nicht_, D‐IAT_angenehm/unangenehm_, D‐IAT_Ich mag/Ich mag nicht_). We used the RM‐ANOVA over t‐tests in the first step to simultaneously examine the effects of both group and D‐IAT versions, as well as their interaction, while accounting for the correlations between the different versions within subjects. In the second step, we subsequently analyzed each D‐IAT version separately, applying one‐tailed independent samples t‐tests, consistent with previous D‐IAT research (Millner et al. 2018; Nock, Park, et al. 2010; Rath et al. 2018). Being aware that this approach increases the risk of family‐wise error due to multiple comparisons, we applied False Discovery Rate (FDR) correction as proposed by Benjamini and Hochberg (1995) to control for the false discovery rate. This method offers a higher correlation between raw and FDR‐adjusted *p*‐values compared to other pairwise combinations, effectively reducing both false positives and false negatives. The adjusted *p* values provide a more conservative estimate of significance while maintaining statistical rigor in the interpretation of group differences across the D‐IAT versions (Jafari and Ansari‐Pour 2019).

As mental disorders, such as depressive (Brådvik 2018; Nock, Hwang, et al. 2010) and anxiety disorders (Wiebenga et al. 2021), especially GAD (de Beurs et al. 2019), are often correlated with suicidal ideation, we conducted hierarchical logistic regression analyses to examine the incremental predictive validity of the D‐values of each D‐IAT version on suicidal ideation, controlling for age, gender, and diagnosis of depressive and anxiety disorders. Furthermore, receiver operating characteristic (ROC) curves were calculated using D‐values of all three D‐IAT versions to determine the discriminant accuracy in distinguishing between suicide ideators and clinical controls. Area under the curve (AUC) values, SEs, and *p* values were computed to quantify the performance of each version in distinguishing between the groups.

Following our third aim, correlations between mean D‐values of all D‐IAT versions and the mean scores of the BSSI were calculated across the total sample to investigate the implicit‐explicit association of implicit attitudes towards death and self‐reported suicidal ideation. A power analysis of a two‐tailed test for significant correlations under a bivariate normal model with a medium effect size (0.3), a power of 0.8, and a significance level (*α*) less than 0.05 was performed, indicating a required sample size of at least *N* = 84. Hence, our sample was sufficiently powered. Finally, we calculated the split‐half reliability to determine the internal validity by correlating the scores from Blocks 6/3 and 7/4, following the standard procedure outlined by Schnabel et al. (2008).

## 5 Results

### 5.1 Descriptive Statistics

Descriptive statistics of the three versions of the D‐IAT can be found in Table 5. Overall, based on the mean statistics, the D‐IAT_me/not me_ exhibited the weakest negative D‐values.

**TABLE 5:** Descriptive statistics for all D‐IAT versions.

	Suicide ideators (*n* = 91)		Clinical controls (*n* = 91)		Test statistic	*p* value	FDR adjusted *p* value	Effect size
	M (SD)	95% CI	M (SD)	95% CI				
D‐IAT_me/not me_	−0.25 (0.27)	[−0.30, −0.19]	−0.35 (0.31)	[−0.42, −0.29]	2.51^c^	0.007	0.021	0.29
D‐IAT_pleasant/unpleasant_	−0.60 (0.32)	[−0.66, −0.53]	−0.65 (0.35)	[−0.72, −0.57]	0.99^c^	0.162	0.162	0.33
D‐IAT_I like/I don’t like_	−0.60 (0.30)	[−0.66, −0.54]	−0.70 (0.33)	[−0.77, −0.63]	2.20^c^	0.015	0.023	0.31

Abbreviations: M = mean; SD = standard deviation.

^c^ One‐tailed independent samples *t*‐test.

### 5.2 Group Differences in Implicit Associations With Death

Mauchly's test indicated that the assumption of sphericity had been met (*χ* ^2^(2) = 2.84, *p* = 0.242). The within‐subjects effect of “iat_version” was large, *F*(2, 360) = 98.53, *p* < 0.001, *η* ^2^ = 0.35. The between‐subjects effect “group” had a small to medium main effect, *F*(1, 180) = 6.62, *p* = 0.011, *η* ^2^ = 0.04, indicating differences between the suicide ideators and clinical controls. However, we found no interaction between “iat_version” and “group” *F*(2, 360) = 0.68, *p* = 0.509, *η* ^2^ = 0.004.

**FIGURE 2:**
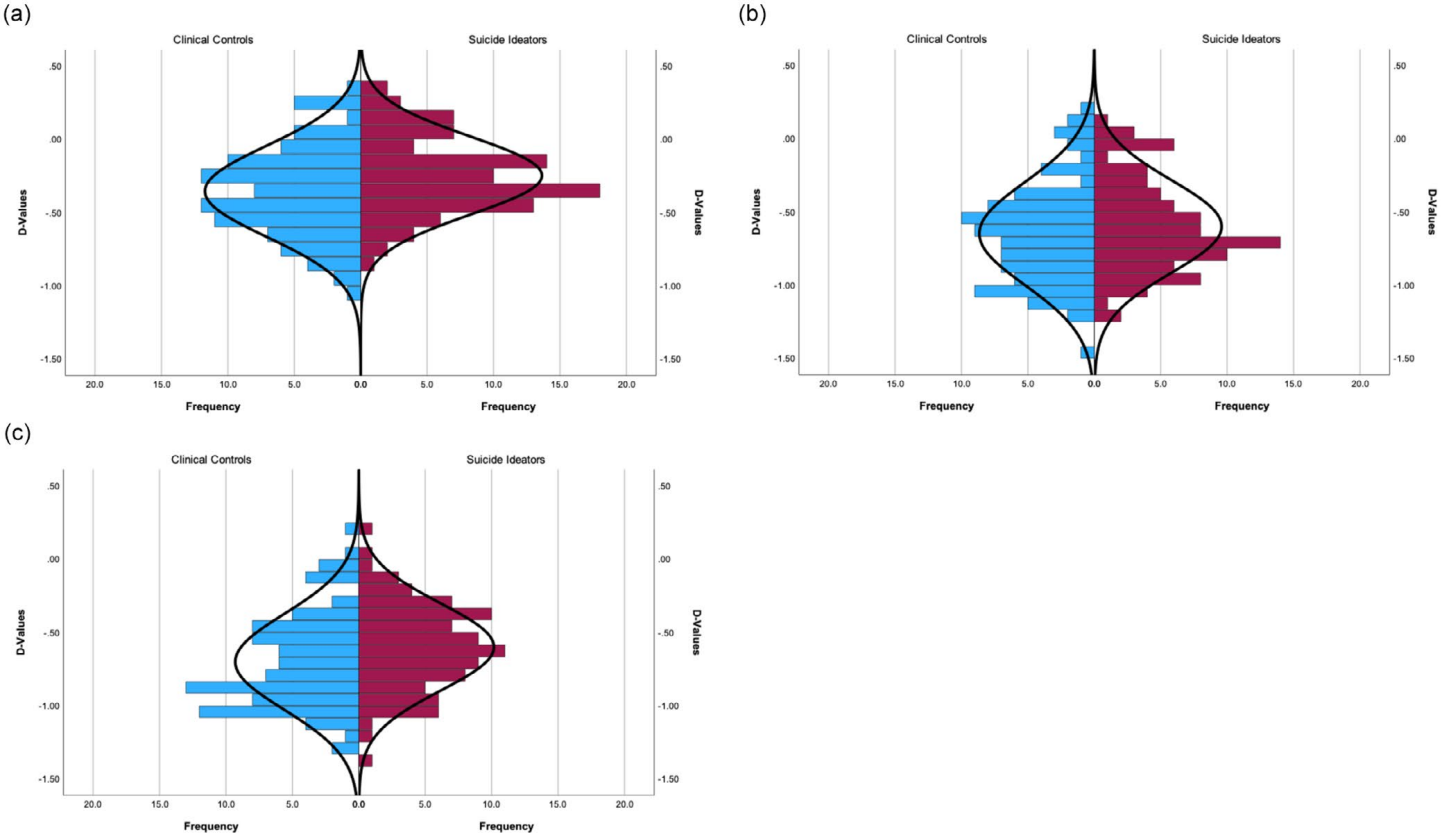
D‐value distributions of all D‐IAT versions in suicide ideators and clinical controls. The D‐values represent the strength of implicit associations between the self and death, as measured in (a) D‐IAT_me/not me_, as well as the positivity of the implicit emotional evaluation of death, with more positive D‐values indicating more positive evaluations, as measured in (b) D‐IAT_pleasant/unpleasant_ and (c) D‐IAT_I like/I don’t like_.

To determine differences in the D‐IAT versions between suicide ideators and clinical controls, separate *t*‐test analyses were conducted for each version. For the standard D‐IAT_me/not me_, a small group effect was observed, *t*(180) = 2.51, *p* = 0.007, *d* = 0.29, indicating differences in the D‐values between suicide ideators and clinical controls. For the D‐IAT_pleasant/unpleasant_, the group effect was not significant, *t*(180) = 0.99, *p* = 0.162, *d* = 0.33. A small group effect was found for the D‐IAT_I like/I don’t like_, *t*(180) = 2.20, *p* = 0.015, *d* = 0.31. As presented in Figure 2, the sample distribution was unaffected by outlier‐driven effects.

After FDR correction, the adjusted *p*‐values were as follows: D‐IAT_me/not me_, *p* = 0.021; for D‐IAT_pleasant/unpleasant_, *p* = 0.162; for D‐IAT_I like/I don’t like_, *p* = 0.023. After adjusting for multiple comparisons, the D‐IAT_me/not me_ and D‐IAT_I like/I don’t like_ remained significant, indicating group differences in implicit associations measured by these versions, that is, suicide ideators demonstrated a stronger association between themselves and death and a more positive liking towards death compared to the clinical controls.

### 5.3 Predictive and Discriminative Validity of Implicit Associations With Death for Suicidal Ideation

For the standard D‐IAT_me/not me_, after controlling for age, gender, and diagnosis of depressive and anxiety disorders, the logistic regression revealed a significant positive association between D‐values and suicidal ideation, *χ* ^2^(1, 182) = 5.84, *p* = 0.016. This indicates that for increasing D‐values, the log odds of experiencing suicidal ideation increase, controlling for age, gender, and diagnosis of depressive and anxiety disorders. For the D‐IAT_pleasant/unpleasant_, the association between D‐value and suicidal ideation was not significant, *χ* ^2^(1, 182) = 0.90, *p* = 0.686. Similarly, for the novel D‐IAT_I like/I don’t like_, the association between D‐value and suicidal ideation did not reach conventional levels of significance, *χ* ^2^(1, 182) = 1.84, *p* = 0.190. When considering all D‐IAT versions simultaneously in the third step of the regression, the D‐value for D‐IAT_me/not me_ remained positively significant, *χ* ^2^(1, 182) = 5.09, *p* = 0.024. This model showed moderate explanatory power (*R* ^*2*^ = 0.28). Additionally, younger age was consistently associated with increased odds of suicidal ideation across all models (*p*s < 0.007), while gender was not a significant predictor in any of the models (*p*s > 0.298). Moreover, the presence of major depression was consistently associated with increased odds of suicidal ideation (*p*s < 0.001) across all models.

For the standard D‐IAT_me/not me_, the AUC was 0.60 (SE = 0.04, *p* = 0.018, CI [0.52, 0.68]). For the D‐IAT_pleasant/unpleasant_, the AUC was 0.54 (SE = 0.04, *p* = 0.337, CI [0.46, 0.63]). For D‐IAT_I like/I don’t like_, the AUC was 0.61 (SE = 0.04, *p* = 0.011, CI [0.53, 0.69]). Overall, all versions showed poor discriminative ability (Hosmer and Lemeshow 2000) in identifying suicide ideators.

### 5.4 Relationship Between Implicit Association With Death and Explicit Suicidal Ideation

We first evaluated convergent validity, examining correlations between the D‐values obtained from the different versions. The results revealed significant positive correlations between D‐IAT_me/not me_ and D‐IAT_pleasant/unpleasant_ (*r*(180) = 0.24, *p* = 0.001, 95% CI [0.10, 0.37]) as well as between D‐IAT_me/not_ me and D‐IAT_I like/I don’t like_ (*r*(180) = 0.21, *p* = 0.005, 95% CI [0.07, 0.34]), indicating weak to moderate associations between the attitude and standard D‐IATs. Additionally, a strong positive correlation was found between D‐IAT_I like/I don’t like_ and D‐IAT_pleasant/unpleasant_ *r*(180) = 0.42, *p* < 0.001, 95% CI [0.29, 0.53]. These findings suggest moderate convergent validity among the D‐IAT versions, indicating that they measure related constructs.

Correlations between the D‐values from each D‐IAT version and the BSSI scores across both groups showed mixed patterns. For D‐IAT_me/not me_, there was a significant positive correlation with BSSI scores (*r*(180) = 0.18, *p* = 0.015, 95% CI [0.04, 0.32]), suggesting a small but statistically significant association. Conversely, for D‐IAT_pleasant/unpleasant_, the correlation with BSSI scores was positive but not statistically significant (*r*(180) = 0.12, *p* = 0.100, 95% CI [−0.02, 0.26]). For D‐IAT_I like/I don’t like_, a significant positive correlation with BSSI scores was observed (*r*(180) = 0.26, *p* < 0.001, 95% CI [0.12, 0.39]), indicating a small and statistically significant association.

The split‐half reliability for D‐values of D‐IAT_me/not me_ was 0.45 (*p* < 0.001), for D‐IAT_pleasant/unpleasant_ it was 0.71 (*p* < 0.001), and for D‐IAT_I like/I don’t like_ it was 0.67 (*p* < 0.001).

## 6 Discussion

### 6.1 Exploring Implicit Associations With Death Across Different D‐IAT Versions

The present study explored implicit emotional associations with death in patients currently experiencing suicidal ideation using two novel attitude‐based versions of the D‐IAT and compared them to the standard identity version. Our primary objective was to discern differences in these associations between suicide ideators and clinical controls. The standard identity version of the D‐IAT outperformed the novel attitude versions in differentiating between the studied groups. To a smaller degree, the D‐IAT_I like/I don’t like_, applying personalized category labels, also showed differences, which indicates distinctive associations related to personal preferences. This pattern parallels findings in addiction research where personalized IATs showed stronger implicit associations with alcohol in heavy drinkers when extrapersonal contamination was reduced (Houben and Wiers 2007). Interestingly, all D‐IAT versions indicated that suicide ideators exhibit weaker negative implicit associations with death instead of stronger positive associations, reflecting a relative ambivalence. Therefore, it is not clear if they reflect a reduced aversion to death or a reduced propensity for life in suicidal ideation. These findings are in line with the existing literature that postulates breaking down D‐IAT versions, especially the interpretation of the D‐values on associations with life versus death (O'Shea et al. 2020).

### 6.2 Predictive and Discriminant Validity

We further explored the predictive and discriminant validity of our attitude versions compared to the identity version. The identity‐based D‐IAT_me/not me_ enhanced the statistical prediction of suicidal ideation, even after controlling for demographic and psychiatric factors. Conversely, the attitude D‐IATs did not identify key factors contributing to suicidal ideation, indicating potential limitations in their incremental predictive utility. These findings echo previous research by Nock and Banaji (2007), who found stronger predictive effects for identity‐based implicit assessments compared to attitude‐based versions, underscoring the importance of considering the conceptual underpinnings of implicit associations in clinical assessments. The discriminative validity performance across the versions was mixed, with the D‐IAT_me/not me_ and D‐IAT_I like/I don’t like_ demonstrating fair discriminative ability in identifying individuals with suicidal ideation.

### 6.3 Relationships Between Implicit and Explicit Measures of Suicidal Ideation

Previous research has highlighted the limitations of explicit self‐report measures in assessing risk or the presence of ideation, as many patients do not disclose their thoughts (Vannoy and Robins 2011). These traditional suicide risk assessment methods also face challenges due to the fluctuating nature of suicidal ideation (Hallensleben et al. 2018; Hawton et al. 2022). Our study addressed this limitation by exploring an assessment that is subject to little conscious influence (Greenwald and Banaji 1995), may access information that is not readily available through self‐report (St Quinton and Brunton 2017) and is less susceptible to the fluctuations observed in suicidal ideation (Hallensleben et al. 2018). Our findings, showing small but positive links between the identity D‐IAT_me/not me_ and the attitude D‐IAT_I like/I don’t like_ with self‐reported suicidal ideation, provide new perspectives by tapping into implicit attributive and emotional processes related to the concept of death. The slightly stronger association observed with the attitude D‐IAT_I like/I don’t like_ implies that personal emotional evaluations may play a role in perceived suicidal ideation, offering complementary insights to the identity‐based approach. In contrast, the attitude D‐IAT_pleasant/unpleasant_ showed no such links to suicidal ideation. The relationship between our implicit and explicit measures aligns with previous research suggesting an implicit‐explicit link in suicidal ideation (Freichel and O'Shea 2023). Thus, the D‐IAT may reveal aspects of suicidal ideation that individuals may not be fully aware of or able to articulate.

Our findings of the split‐half reliability analyses align with those described by Rath et al. (2021) and Millner et al. (2019) for the D‐IAT, whose studies reported reliabilities of 0.59 and 0.65 to 0.69, and Nosek et al. (2007), for the IAT (0.7–0.9). Specifically, the adapted attitude versions demonstrated reliability similar to or higher. The reliability of D‐IAT_me/not me_ was lower, suggesting potential limitations in its measurement precision.

### 6.4 Overall Performance Differences of the Different D‐IAT Versions

Consistent with prior research exploring the multifactorial underpinnings of suicide risk (Borges et al. 2010; Glenn et al. 2017), our observations highlight the need for a multidimensional understanding (see also Orsolini et al. 2020). While the identity D‐IAT overall performed superiorly, specifically the novel version with personalized categories provides an opportunity to assess distinctive patterns characterized by a positively valenced implicit emotional association with death. The D‐IAT_I like/I don’t like_ has demonstrated group differences and validity in the context of correlations with self‐reported suicidal ideation. This version has demonstrated discriminative accuracy similar to the identity version and a trend in predictive validity, suggesting that the “I like/I don't like” paradigm outperforms the “pleasant/unpleasant” version. Thus, our study adds a novel emotional dimension to the current understanding of the implicit mechanisms associated with suicidal ideation.

## 7 Clinical Implications

Clinicians should consider the significance of implicit cognitive biases, particularly positively valenced associations with death observed in current suicidal ideation. While the current cross‐sectional data do not allow for comprehensive insights into the dynamics of suicidality or prognostic determinations, they highlight the relevance of implicit processes during the stage of suicidal ideation. Understanding this critical stage is crucial in the clinical context, as it precedes the transition to behavior, as proposed by Klonsky and May (2014).

Given the persistence of implicit biases towards suicide even after the resolution of suicidal ideation (Wells et al. 2020), integrating interventions that target these implicit patterns could potentially augment suicide prevention strategies. Although interventions aimed at modifying implicit biases have produced mixed results (FitzGerald et al. 2019; Cha et al. 2017), evidence from conditioning paradigms indicates that biased cognitive processing can be altered (Clerkin and Teachman 2010). Interventions aimed at overriding racial (Calanchini et al. 2021) and addiction‐related (Copersino 2017) biases were found to influence implicit associations and control‐oriented processes.

In conclusion, while explicit self‐report measures remain indispensable, incorporating implicit measures could offer a more comprehensive understanding of an individual's risk profile.

## 8 Implications for Future Studies

Despite the reliable meta‐analytic effects of the D‐IAT (Sohn et al. 2021), the variable results observed in studies using the D‐IAT, including our results, highlight the need for continued research into implicit associations. Future investigations should systematically explore factors contributing to this variability, such as specific characteristics of the groups studied, especially those exhibiting suicidal tendencies, so that findings across studies can be interpreted and compared. Additionally, assessing distinctions in other variables, such as cultural differences, diverse clinical profiles, and methodological approaches, may contribute to establishing more consistent patterns in implicit associations related to suicide.

While research typically focuses on suicidal behaviors due to their significance and potential for substantial between‐group effects (Kleiman 2020), further development of attitude‐related D‐IATs that assess personal emotions in suicidal ideation could enable early detection of potential risks. Gysin‐Maillart et al. (2022) found that understanding the reasons for death rather than those for life may be particularly salient for achieving therapeutic efficacy. Examining the specific mechanisms driving positive evaluations of death may offer new ways of understanding and addressing suicidal ideation from both scientific and clinical perspectives.

Further research is necessary to refine and validate the adapted tasks introduced in our study, focusing on comprehensive validation across diverse populations and the utilization of longitudinal designs. Longitudinal studies could offer insights into the dynamic nature of these emotional associations over time and could examine whether the D‐IAT functions as a behavioral marker or an enduring aspect of identity.

## 9 Limitations

While we studied subjects with current suicidal ideation using novel D‐IAT versions, several limitations should be considered when interpreting the findings. First, we recruited inpatients with a narrow set of psychiatric diagnoses, limiting generalizability. Second, we had to exclude a number of datasets due to participants' inability to perform the task or diagnostic restrictions. While this procedure refined the data analysis, it concurrently introduced further selection bias. Finally, we ran a substantial number of group comparisons. In order to minimize type II errors, we corrected the post hoc tests for multiple comparisons using the FDR method.

## 10 Conclusion

Our study confirms the validity of the standard identity D‐IAT as a predictor of suicidal ideation while offering a complementary adapted attitude version using personalized affective categories. We found evidence of implicit emotional associations linking death to personal liking in patients currently experiencing suicidal ideation, highlighting the potential of supplementing the identity assessment with personalized attitude variants. Moving forward, comprehensive development and validation of implicit emotional measures, including personalized variants, is crucial for enhancing suicide risk assessment and prevention strategies.

## Author Contributions

**L. M. Aschenbrenner:** data curation (lead), formal analysis (lead), investigation (equal), project administration (equal), visualization (lead), writing – original draft (lead). **A. Frei:** data curation (supporting), investigation (equal), project administration (supporting), writing – review and editing (equal). **D. Knapp:** investigation (supporting), writing – original draft (supporting). **T. Forkmann:** conceptualization (equal), methodology (equal), validation (equal), writing – review and editing (equal). **D. Schreiber:** conceptualization (equal), methodology (equal), validation (equal), writing – review and editing (equal). **H. Glaesmer:** conceptualization (equal), methodology (equal), validation (equal), writing – review and editing (equal). **J. Brüdern:** conceptualization (equal), methodology (equal), validation (equal), writing – review and editing (equal). **M. Stein:** methodology (equal), validation (equal), writing – review and editing (equal). **S. Walther:** funding acquisition (lead), resources (lead), software (equal), supervision (equal), writing – review and editing (equal). **A. Gysin‐Maillart:** conceptualization (lead), funding acquisition (equal), investigation (equal), methodology (equal), project administration (equal), resources (equal), supervision (equal), validation (equal), writing – review and editing (lead).

## Conflicts of Interest

The authors declare no conflicts of interest.
